# Autism spectrum disorder and Li-Fraumeni syndrome: purely coincidental or mechanistically associated?

**DOI:** 10.1186/s40348-017-0075-9

**Published:** 2017-10-05

**Authors:** Michaela Kuhlen, Julia Taeubner, Dagmar Wieczorek, Arndt Borkhardt

**Affiliations:** 1 Department of Pediatric Oncology, Hematology and Clinical Immunology, University Children’s Hospital, Medical Faculty, Heinrich Heine University, Moorenstr. 5, 40225 Duesseldorf, Germany; 2 Institute of Human Genetics, Medical Faculty, Heinrich Heine University, Duesseldorf, Germany

**Keywords:** Autism spectrum disorder, Li-Fraumeni syndrome, Hypodiploid leukemia

## Abstract

**Background:**

Autism spectrum disorders (ASDs) are neurodevelopmental disorders with impaired social interactions and communication and restrictive, repetitive patterns of behaviors, interests, and activities. A recent epidemiological study suggests that children with ASD might have an increased cancer risk.

**Case presentation:**

The 14.5-year-old boy, previously diagnosed with ASD, was referred with persistent bone pain. Diagnostic work-up confirmed diagnosis of acute lymphoblastic leukemia (ALL); cytogenetic analysis revealed low hypodiploid karyotype with a mutation (c.733G>A, p.Gly245Ser, rs28934575) in *TP53* in the leukemic blasts. By Sanger sequencing, the presence of this mutation in the germline was subsequently confirmed and, thus, diagnosis of Li-Fraumeni syndrome (LFS) was made. His family history was remarkable with two siblings with intellectual disability and a mother who has died of premenopausal breast cancer.

**Conclusions:**

Some of the oncogenes and tumor suppressor genes causing cancer susceptibility syndromes overlap with those involved in autism. This functional overlap between autism and cancer is novel and particularly compelling. The surprising coincidence of LFS and ASD in our patient raises the question whether this is purely coincidental or mechanistically associated.

## Findings

Autism spectrum disorder (ASD) is a group of neurodevelopmental disorders with impaired social interactions and communication and restrictive, repetitive patterns of behaviors, interests, and activities. The prevalence of ASDs in the general population has been estimated to be 1% worldwide, with a high male to female ratio. This number seems even higher in children with around 1 in every 68 children (Report 2014 of *The Centers for Disease Control and Prevention’s Autism and Developmental Disabilities Monitoring Network*). A recent epidemiological study suggests that children with ASD might have an increased cancer risk [1].

We report a boy of non-consanguineous parents, previously diagnosed with ASD at the age of 13 years. His family history was remarkable with an older sister with intellectual disability not further specified, a younger brother with profound and multiple disabilities, and a mother who has died of premenopausal breast cancer. The boy was referred to our department at the age of 14.5 years with persistent bone pain. MRI revealed leukemic infiltration of the femura and spine, and further diagnostic work-up confirmed the diagnosis of ALL. Cytogenetic analysis revealed a low hypodiploid karyotype with a deletion of 17p and molecular analysis an additional mutation (c.733G>A, p.Gly245Ser, rs28934575) in *TP53* (NM_000546.5) in the leukemic blasts. The mutation was subsequently confirmed by conventional Sanger sequencing in fibroblast-derived germline DNA; thus, the diagnosis of Li-Fraumeni syndrome (LFS, OMIM #151623) was made. The boy was treated according to the high-risk protocol of the AIEOP-BFM 2009 trial and is, 24 months after diagnosis, doing well under maintenance therapy.

This report documents the surprising coincidence of LFS and ASD, thus, raising the question whether this is purely coincidental or mechanistically associated.

A high number of ASDs are hereditary cases [2], and there is mounting evidence that childhood cancer has likewise a higher fraction of cases with genetic traits than previously expected [3].

Advances in high-throughput sequencing have led to greater understanding of genetic mechanisms in ASD. Several of the susceptibility genes identified in ASD patients that are also associated with cancer are involved in chromatin remodeling, genome maintenance, signal transduction pathways, histone modification, and share functions as transcription factors [4, 5]. Some of the oncogenes and tumor suppressor genes like *PTEN* and *NF1* are implicated in causing hereditary cancer susceptibility syndromes overlap with those involved in autism [4, 6]. Furthermore, copy number variations (CNV) recently described in children with autism are associated with cancer predisposition genes. This functional overlap between autism and cancer is novel and particularly compelling.

Li-Fraumeni syndrome, resulting from germline mutations in the *TP53* gene, represents a well-known cancer susceptibility syndrome. *TP53* is a tumor suppressor gene encoding the transcription factor p53 that responds to several forms of cellular stress and, thus, plays a pivotal role in cell growth control, DNA repair, cell cycle suppression and senescence and eventually in the initiation of apoptosis. Experimental assays on the functional impact of mutant proteins have revealed different mechanisms including (1) transactivation of reporter genes in the (downstream) pathways of p53; (2) activation of genes that are independent, unrelated to, and/or repressed by the wild-type protein (gain of function; GOF); (3) induction of cell cycle arrest or apoptosis; and (4) dominant-negative effect over the wild-type protein.

Interestingly, recent studies imply, on the one hand, a negative feedback loop between *TP53* and phosphatase and tensin homolog (*PTEN*) [7–9] and, on the other hand, a link between autism and incomplete loss of *PTEN* function [10]. Additionally, it was also shown that *p53* gene copy ratios were increased in children with autism [11]. It might be hypothesized that ASD is mechanistically associated with LFS and, thus, might be part of the LFS phenotype.

We observed a surprising coincidence of LFS with ASD, which warrants further testing in large cooperative studies.

## Abbreviations

ALL: Acute lymphoblastic leukemia
ASD: Autism spectrum disorder
CNV(s): Copy number variant(s)
LFS: Li-Fraumeni syndrome
PTEN: Phosphatase and tensin homolog
